# Modified vaccinia virus Ankara expressing the hemagglutinin of pandemic (H1N1) 2009 virus induces cross-protective immunity against Eurasian ‘avian-like’ H1N1 swine viruses in mice

**DOI:** 10.1111/irv.12221

**Published:** 2013-12-23

**Authors:** Maria R Castrucci, Marzia Facchini, Giuseppina Di Mario, Bruno Garulli, Ester Sciaraffia, Monica Meola, Concetta Fabiani, Maria A De Marco, Paolo Cordioli, Antonio Siccardi, Yoshihiro Kawaoka, Isabella Donatelli

**Affiliations:** a Department of Infectious, Parasitic and Immune-Mediated Diseases, Istituto Superiore di SanitàRome, Italy; b Department of Biology and Biotechnology “Charles Darwin”, University of Rome “La Sapienza”Rome, Italy; c Institute for Environmental Protection and Research, Ozzano Emilia (BO)Bologna, Italy; d Istituto Zooprofilattico Sperimentale della Lombardia e dell'Emilia RomagnaBrescia, Italy; e San Raffaele Scientific InstituteMilano, Italy; f Department of Pathobiological Sciences, School of Veterinary Medicine, University of Wisconsin-MadisonMadison, WI, USA; g Division of Virology, Department of Microbiology and Immunology, International Research Center for Infectious Diseases, Institute of Medical Science, University of TokyoTokyo, Japan; h Infection-Induced Host Responses Project, Exploratory Research for Advanced TechnologySaitama, Japan

**Keywords:** Avian-like, cross-protection, hemagglutinin, influenza, pandemic (H1N1) 2009, transmission

## Abstract

**Objectives:**

To examine cross-reactivity between hemagglutinin (HA) derived from A/California/7/09 (CA/09) virus and that derived from representative Eurasian “avian-like” (EA) H1N1 swine viruses isolated in Italy between 1999 and 2008 during virological surveillance in pigs.

**Design:**

Modified vaccinia virus Ankara (MVA) expressing the HA gene of CA/09 virus (MVA-HA-CA/09) was used as a vaccine to investigate cross-protective immunity against H1N1 swine viruses in mice.

**Sample:**

Two classical swine H1N1 (CS) viruses and four representative EA-like H1N1 swine viruses previously isolated during outbreaks of respiratory disease in pigs on farms in Northern Italy were used in this study.

**Setting:**

Female C57BL/6 mice were vaccinated with MVA/HA/CA/09 and then challenged intranasally with H1N1 swine viruses.

**Main outcome measures:**

Cross-reactive antibody responses were determined by hemagglutination- inhibition (HI) and virus microneutralizing (MN) assays of sera from MVA-vaccinated mice. The extent of protective immunity against infection with H1N1 swine viruses was determined by measuring lung viral load on days 2 and 4 post-challenge.

**Results and Conclusions:**

Systemic immunization of mice with CA/09-derived HA, vectored by MVA, elicited cross-protective immunity against recent EA-like swine viruses. This immune protection was related to the levels of cross-reactive HI antibodies in the sera of the immunized mice and was dependent on the similarity of the antigenic site Sa of H1 HAs. Our findings suggest that the herd immunity elicited in humans by the pandemic (H1N1) 2009 virus could limit the transmission of recent EA-like swine HA genes into the influenza A virus gene pool in humans.

## Introduction

Following its appearance in 2009, a novel reassortant H1N1 virus of swine origin quickly spread worldwide, causing the first influenza pandemic of the 21st century. Thereafter, infections with the pandemic (H1N1) 2009 virus (pdm/09) were reported in pigs and other species.^1^–^4^ In particular, the introduction of virus from humans to swine on multiple independent occasions and the cocirculation of strains from different hosts in these animals, which act as ‘mixing vessels’ to facilitate reassortment, raise concerns about the possible emergence of further novel viruses of potential threat to humans.^5^–^9^ In this context, a new variant virus of swine origin (H3N2v) containing the pdm/09 M gene has been isolated from human cases in the USA since July 2011. This virus is antigenically distinct from the human seasonal H3N2 viruses circulating worldwide, and most cases have been in young children who had contact with pigs.^10^ Moreover, in southern China, cocirculation of viruses belonging to three major lineages of swine H1 influenza viruses, that is, classical swine H1N1 (CS), Eurasian ‘avian-like’ H1N1 (EA), and triple reassortant H1N2 viruses, has already afforded opportunities for the generation of novel reassortants with EA-like swine H1N1 surface genes and pdm/09 internal genes.^11^,^12^ These novel viral reassortants have been isolated from pigs, and their potential establishment and efficient transmission from pig to pig could provide opportunities for human infections.

Although the EA-like viruses have been prevalent in Eurasian pig populations for more than 30 years, most humans are immunologically naïve to EA-like viruses, with the exception of individuals with occupational exposure to pigs.^13^,^14^ However, seroconversion to pdm/09 virus has been associated with cross-reactive immunity to other swine influenza viruses, including EA-like swine viruses.^15^,^16^ Furthermore, cross-reactivity between the pdm/09 virus and EA-like viruses, as well as with A/New Jersey/8/76 (NJ/76) virus, has been detected in pig serum samples examined in different infection and vaccination studies.^17^,^18^ Studies designed to investigate the antigenic characteristics of influenza viruses circulating in pigs and to assess specific immune reactivities in humans may thus help define the conditions that would permit the introduction of new HA genes into the gene pool of human influenza viruses.

Here, we used replication-deficient recombinant modified vaccinia virus Ankara (MVA) expressing the HA gene of A/CA/07/09 (CA/09) virus as a vaccine to investigate cross-reactivity to representative EA-like swine viruses isolated during virologic surveillance studies of influenza viruses in pigs in Italy. We also assessed the ability of this vaccine to protect mice against viral challenge.

## Methods

### Viruses

The A/sw/It/2034/1999 (sw/It/99), A/sw/It/206919-2/2002 (sw/It/02), A/sw/It/232868/2007 (sw/It/07), and A/sw/It/207871/2008 (sw/It/08) H1N1 viruses were previously isolated during outbreaks of respiratory disease in pigs on farms in northern Italy. A/sw/Iowa/15/30 (sw/IA/30) and NJ/76 were included in the study as representative classical swine H1N1 (CS) influenza viruses. The A/California/7/09 (CA/09) (H1N1) virus was provided by the Centers for Disease Control and Prevention (CDC). The viruses were propagated in embryonated chicken eggs. Stock virus titers were determined both through HA titration using 0·5% turkey erythrocytes and by the calculation of the fifty percent tissue culture infectious dose (TCID_50_) in MDCK cells.^19^

### Antigenic characterization

For the antigenic characterization of the Italian swine viruses, hyperimmune chicken antisera against reference strains were used in the hemagglutination-inhibition (HI) assay, according to previously described methods.^19^

### Nucleotide sequence analysis

Viral RNA was extracted from allantoic fluid, and RT-PCR amplification and HA gene sequence reactions were carried out as described elsewhere.^20^ DNA sequences were analyzed using the Lasergene package (version 4.0; dnastar, Inc., Madison, WI, USA). Phylogenetic analyses were conducted with mega4 software (The Biodesign Institute, Arizona State University, Tempe, AZ, USA). Swine and human influenza virus sequences published in GenBank database were included in the multiple alignments. Nucleotide sequences obtained in this study are available upon request.

### Construction of recombinant MVA

Recombinant MVA expressing the HA gene of CA/09 virus (MVA-HA-CA/09) was prepared as previously described.^21^ Serum-free cultures of chicken embryo fibroblasts were utilized for recombinant virus construction, terminal dilution cloning, and virus stock production.

### Vaccination of mice and virus challenge

Female C57BL/6 mice, 6–8 weeks old, were vaccinated intramuscularly (i.m.) with two doses of 10^4·3^ to 10^7·3^ pfu of MVA-HA-CA/09 (*n* = 6/group), administered 3 weeks apart. Four weeks after the last immunization, mice were anesthetized with avertin and challenged intranasally (i.n.) with 100 50% lethal dose (LD_50_) (corresponding to 1·6 × 10^4^ TCID_50_) of the CA/09 virus in a 50 μl volume. Naïve mice were included as a negative control. Mice were then monitored for survival for 21 days after infection, and those with signs of severe disease and weight loss of >25% were humanely killed. Animal experiments were performed in compliance with institutional guidelines and approved protocols.

For the challenge with swine viruses, groups of mice vaccinated with two doses of MVA containing 10^7·3^ or 10^6·3^ pfu were infected i.n., 4 weeks later, with 50 μl of 10^5^ TCID_50_ of one of the following viruses: sw/IA/30, NJ/76, sw/It/99, sw/It/02, sw/It/07, or sw/It/08. Control mice received an equal volume of PBS. On day 2 and 4 post-infection (p.i), the lungs from four mice in each group were harvested, and lung homogenates were titrated in MDCK cells.

### Animal serology

Serum samples were collected from mice vaccinated with MVA-HA-CA/09 virus immediately before challenge, and used in HI and virus microneutralizing (MN) assays. Sera were treated overnight with receptor-destroying enzyme (RDE) at 37°C and heat inactivated at 56°C for 45 minutes.^19^ Virus-neutralizing titers were determined by infection of MDCK cells and are expressed as the reciprocal of the highest dilution of serum that gave complete neutralization of 100 TCID_50_ of virus after incubation at 37°C for 72 hours.

Hyperimmune sera obtained from mice immunized intraperitoneally twice with 10^6^ TCID_50_ of the swine viruses were used against CA/09 virus in HI and MN cross-reactivity assays.

## Results

### Antigenic and genetic analysis of Italian swine H1N1 viruses

The swine viruses selected for this study were representative of influenza viruses that were isolated from respiratory outbreaks in pigs in Italy between 1999 and 2008. The results of HI tests using post-infection chicken antisera specific to human and swine H1N1 and H3N2 subtype viruses showed that the HA of these swine viruses was antigenically related to the HA of EA-like swine H1N1 viruses (Table S1). In particular, sw/It/99 virus was most closely related to the earlier EA-like reference strain A/sw/Finistere/2899/82, whereas the sw/It/02, sw/It/07, and sw/It/08 viruses were antigenically similar to the more recent A/sw/IV/1455/99-like H1N1 swine viruses (e.g., A/sw/Italy/125746/05 virus) as appears to be the case for most EA-like viruses isolated in Italy (P. Cordioli, unpublished data).

**Figure 1:**
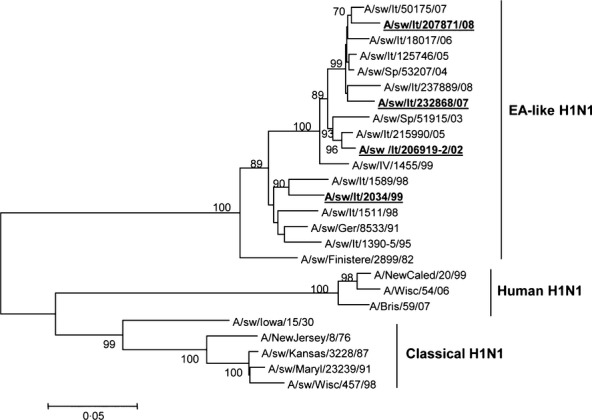
Phylogenetic tree of the HA1 region (nucleotides 88–981) of the HA gene of the H1N1 swine influenza viruses used in this study (indicated in bold and underlined) as well as that of the swine and human influenza viruses in the database. The unrooted tree was generated with the mega4 program using the neighbor-joining method. Bootstrap values were calculated on 1000 replicates, and only values higher 70% are shown.

Phylogenetic analysis of the partial HA gene sequences (nucleotides 88–981) coding for the HA1 region (amino acid residues 30–327) of the influenza viruses isolated in Italy and those available in GenBank confirmed the relationship between the Italian viruses and the EA lineage. In particular, viruses isolated between 2002 and 2008 were located together in the phylogenetic tree in a closely differentiated branch within the EA cluster (Figure 1).

### Serologic cross-reactivity between swine H1 viruses and pandemic H1N1 2009

To determine the extent of antigenic similarity between the swine viruses and CA/09 virus, as a reference strain of pdm/09 viruses, hyperimmune sera from mice experimentally injected with the above EA-like viruses, along with the sw/IA/30 and NJ/76 viruses, as representatives of the CS lineage of swine viruses, were used in HI and MN assays. The mean reciprocal titers for each pair are summarized in Table 1. All antisera displayed high mean HI and MN titers against the homologous viruses with the exception of sw/IA/30, which showed a moderate mean reciprocal HI titer of 160.

**Table 1:** Serologic cross-reactivity between antiswine influenza sera and CA/09 virus

Antiserum*	Homologous		CA/09	
	
	HI	MN	HI	MN
NJ/76	320	1280	40	160
sw/IA/30	160	5120	20	80
sw/It/99	640	5120	<10	40
sw/It/02	320	5120	80	640
sw/It/07	1280	2560	160	1280
sw/It/08	320	2560	160	1280
CA/09			640	5120

Virus abbreviations: A/California/7/09 (CA/09); A/New Jersey/8/76 (NJ/76); A/sw/Iowa/15/30 (sw/lA/30); A/sw/lt/2034/99 (sw/It/99); A/sw/It/206919-2/02 (sw/It/02); A/sw/It/232868/07 (sw/It/07); and A/sw/It/207871/08 (sw/It/08).

* Hyperimmune sera obtained from mice immunized twice with the indicated viruses.

The sera from mice vaccinated with the CS strains were weakly cross-reactive with CA/09 virus in the HI assay, whereas in the MN assay, higher levels of neutralizing activity, relative to the HI activity, were detected in the sera from mice vaccinated with NJ/76. These results suggest that antigenic determinants in HA, other than those recognized by the HI antibodies, contribute to the induction of cross-reactive neutralizing antibodies.

The sera from mice vaccinated with the EA-like virus sw/It/99 gave low or undetectable levels of cross-reactivity with CA/09 virus, in the MN and HI assays, respectively. The highest antigenic cross-reactivity was with antisera from the EA-like viruses isolated in 2007 and 2008 and, to a slightly lesser extent, with the EA-like virus isolated in 2002. Thus, all three of the swine EA-like viruses isolated in Italy between 2002 and 2008 were cross-reactive to CA/09 virus in both the HI and MN assays, in agreement with the serologic cross-reactivity previously observed between swine H1N1 and pdm/09 viruses.^15^,^16^

### Immunogenicity and protection of MVA-HA-CA/09 vaccine against the homologous pdm/09 virus in mice

To determine whether an HA-based vaccine could elicit cross-protective immunity against the EA-like swine viruses, an MVA-HA-CA/09 virus vaccine was generated and its immunogenicity determined in mice. Groups of mice were injected twice with different doses of the vaccine and then challenged with 100 MLD_50_ of the homologous CA/09 virus. All mice that received PBS or 10^7·3^ pfu of MVA-wt experienced rapid weight loss and succumbed to their infection before day 12. Mice vaccinated with 10^7·3^ or 10^6·3^ pfu of MVA-HA-CA/09 were fully protected and showed no clinical signs after challenge (Table 2). Mice vaccinated with 10^5·3^ pfu showed 10% weight loss and 91·6% survival, whereas partial protection (33%) was achieved with 10^4·3^ pfu of MVA-HA-CA/09. The high levels of antibodies induced after immunization of mice with MVA-HA-CA/09 and detected in the pre-challenge sera by the use of HI and MN assays correlated with the survival outcome after lethal challenge with CA/09 virus described above (Table 2).

**Table 2:** Induction of antibodies after immunization with MVA-HA-CA/09 and protection from lethal challenge with CA/09 virus

Vaccine	Dose (pfu)	Titer* (pre-challenge)		Survival after challenge** n/nt*** (%)

		HI	MN	
MVA-HA-CA/09	10^7·3^	320	2560	12/12 (100)
	10^6·3^	320	1280	12/12 (100)
	10^5·3^	160	1280	11/12 (91)
	10^4·3^	20	80	4/12 (33)
MVA-wt	10^7·3^	<10	<10	0/12 (0)
PBS	Mock	<10	<10	0/12 (0)

* Antibody titers of the post-immunization serum pools against CA/09 virus.

** Challenge dose: 100 MLD_50_ of CA/09 virus.

*** n/nt, number of survivors/total animals of two separate experiments with 6 animal per group.

### MVA-HA-CA/09-induced protection against infection with different swine H1 influenza isolates

**Figure 2:**
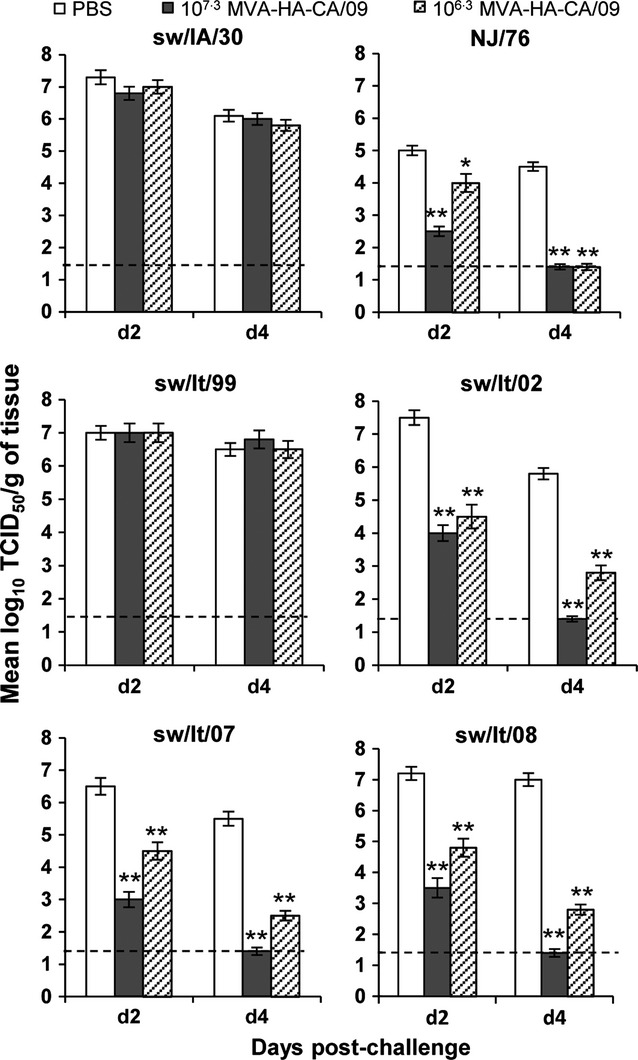
Effect of MVA-HA-CA/09 vaccination on the replication of H1N1 viruses in mouse lung. Groups of mice were vaccinated with two doses, 3 weeks apart, of 10^7·3^ or 10^6·3^ pfu of MVA-HA-CA/09. Control mice received PBS only on the same schedule. Four weeks later, the mice were challenged intranasally with 10^5^ TCID_50_ of the indicated H1N1 viruses. Virus titers in the lungs of four mice per group sacrificed on days 2 and 4 post-infection are expressed as the mean ±SE log_10_TCID_50_/g of tissue. The lower detection limit is denoted by the dashed horizontal line. One of two similar experiments is shown. For virus abbreviations, see Table 1. *P < 0·05, **P < 0·001 compared with control mice by unpaired Student's *t* test.

To establish whether the HA-CA/09-specific immunity could protect against infection with swine H1 viruses, groups of C57BL/6 mice received 10^7·3^ or 10^6·3^ pfu of MVA-HA-CA/09 twice, 3 weeks apart, and were subsequently challenged i.n. with 10^5^ TCID_50_ of sw/IA/30, NJ/76 or the Italian swine viruses. This virus dose led to extensive viral replication in the lungs of unvaccinated mice by days 2 and 4 p.i. for all of the viruses, except for NJ/76 virus, which showed almost 2 log lower titers compared with those of the other viruses (Figure 2). Mice previously vaccinated with MVA-HA-CA/09 and challenged with sw/IA/30 virus had viral loads similar to those observed in the lungs of unvaccinated controls, whereas substantial protection was observed in vaccinated mice at day 4 post-challenge with NJ/76 virus. Among the EA-like viruses, MVA-HA-CA/09-vaccinated mice that were challenged with the earlier sw/It/99 virus showed high viral titers in the lungs, comparable with unvaccinated control mice, whereas a substantial reduction in lung viral load was observed when vaccinated mice were challenged with the sw/It/02, sw/It/07, and sw/It/08 viruses. In particular, mice vaccinated twice with 10^6·3^ pfu of MVA-HA-CA/09 showed more than a 3-log_10_ reduction in virus titers at day 4 p.i., compared with titers in unimmunized control animals, and even better protection was achieved in mice vaccinated with 10^7·3^ pfu of MVA-HA-CA/09 with no virus recovery on day 4 post-challenge.

### Cross-reactive antibody responses induced by immunization with the MVA-HA-CA/09 vaccine

The antigenic relatedness between CA/09 virus and swine H1 viruses was further defined by serologic analyses of pooled sera collected from mice vaccinated twice with MVA-HA-CA-09 before viral challenge. The mean reciprocal titers obtained in the HI and MN assays are summarized in Table 3 and strongly correlated with the results from the *in vivo* challenge experiments. In particular, the sera from mice immunized with 10^7·3^ or 10^6·3^ pfu of MVA/HA-CA/09 did not show detectable levels of HI or MN antibodies when assayed against Sw/IA/30 or Sw/It/99 viruses, in agreement with the complete lack of protection observed in mice challenged with these viruses (Figure 2). In contrast, the sera from the vaccinated mice showed considerable cross-reactivity to NJ/76 and sw/It/02, sw/It/07, and sw/It/08 viruses, and the extent of protection against challenge with these viruses correlated with the levels of cross-reactive antibodies elicited by the two different doses of vaccine. Overall, the presence of cross-reactive antibodies in MVA-HA-CA/09-vaccinated mice correlated closely with their survival outcome after lethal challenge with H1N1 swine viruses. These results suggest that antigenic relatedness exists between the HA of CA/09 virus and the HA of EA-like swine H1 viruses isolated in Italy since 2002.

**Table 3:** Serum cross-reactive antibody response in mice administered MVA-HA-CA/09

Dose (pfu) MVA-HA-CA/09	Serum antibody titer for*													

	NJ/76		sw/lA/30		sw/It/99		sw/It/02		sw/It/07		sw/It/08		CA/09	
						
	HI	MN	HI	MN	HI	MN	HI	MN	HI	MN	HI	MN	HI	MN
10^7·3^	80	160	<10	<10	<10	10	80	160	160	320	80	160	640	2560
10^6·3^	40	80	<10	<10	<10	<10	40	80	80	80	40	80	320	1280

For virus abbreviations, see Table 1.

* Antibody titers of the post-immunization serum pools against the indicated viruses.

### Comparison of the antigenic sites in the HA of H1 swine viruses

**Figure 3:**
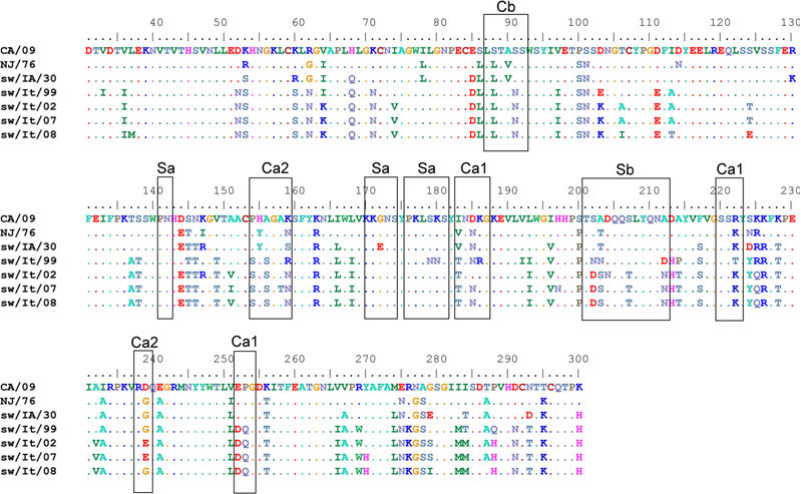
Comparison of the HA antigenic sites. Amino acids sequences (H1 open reading frame numbering, including the signal peptide) of the CS viruses and the Italian swine viruses used in this study were aligned to the CA/09 HA sequence. Major antigenic domains, as defined by Brownlee and Fodor,^22^ are shown in boxes. For virus abbreviations, see Table 1.

To investigate further the molecular basis of the antigenic relationship between the HAs of CA/09 virus and swine viruses, the amino acid sequences of the HA1 region of the Italian swine viruses were aligned and compared with those of CA/09, NJ/76, and sw/IA/30. There was close similarity in the antigenic sites^22^ between NJ/76 and sw/IA/30 viruses, with only two amino acid differences in the antigenic sites Ca2 and Sa at positions 159 and 172, respectively (Figure 3). Notably, the amino acid sequence of the Sa antigenic site of NJ/76 was identical to that of CA/09, which may explain the presence of HI cross-reactive antibodies in mice vaccinated with MVA-HA-CA/09 and their protective efficacy against challenge with NJ/76 virus (Figure 2).

The HA sequences of the sw/It/02, sw/It/07, and sw/It/08 viruses showed similarities at the Sa antigenic site with that in CA/09 virus, and only a small number of single amino acid changes at the antigenic sites Sb, Ca1, and Ca2 were probably responsible for the slight differences in the levels of cross-reactive antibodies measured in the sera of MVA-HA-CA/09-vaccinated mice. In contrast, the HA sequence of the earlier sw/It/99 virus differed the most in its antigenic sites from that of the other EA-like Italian viruses and also from CA/09 virus in antigenic site Sa (S179N, K180N) (Figure 2). Thus, the amino acid residues within antigenic site Sa of CA/09 are conserved among viruses that show cross-protective immunity against NJ/76 virus and the sw/It/02, sw/It/07, and sw/It/08 viruses. Moreover, comparison between these cross-reactive viruses and the EA-like H1N1 swine viruses isolated in Europe over the 13-year period and retrieved in GenBank revealed that 64·61% of the viruses (84 of 130) had sequence homology in the antigenic site Sa (Figure S1), thus suggesting more frequent circulation of cross-reactive than non-cross-reactive H1N1 viruses in pigs.

## Discussion

In this study, we explored the extent of cross-reactivity between HA derived from CA/09 virus and that derived from representative EA-like H1N1 swine viruses isolated in Italy between 1999 and 2008. Our results show that systemic immunization of mice with CA/09-derived HA, vectored by MVA, elicits cross-protective immunity against EA-like virus infection in mice, suggesting that cross-reactivity in humans may also somewhat limit their susceptibility to infection with H1N1 swine viruses of the recent EA lineage.

Several investigators have reported the presence of pre-existing cross-reactive neutralizing antibodies against the pdm/09 virus in individuals over 60 years of age.^23^–^25^ Cross-reactive HI and NI antibodies induced by the 1976 swine flu vaccines against pdm/09 viruses in archived human serum samples collected during the 1976 swine flu vaccine trial and their protective efficacy in mice against pdm/09 virus infection may explain the low mortality associated with pdm/09 virus infection of older adults in the USA^26^ Cross-reactive immune responses between pdm/09 and H1N1 viruses of the CS lineage have also been reported.^27^–^30^ Moreover, evidence that primary infection with NJ/76 and sw/IA/30 viruses provided complete protection from challenge with CA/09 virus in both mice and ferrets further supports the antigenic relatedness among these H1N1 viruses, although cross-reactive T cells can also effectively help blunt disease severity following CA/09 virus infection.^31^–^34^

The HA of EA-like swine viruses is derived from influenza viruses of avian origin that cross-react poorly with human or CS H1N1 viruses. Nevertheless, a recent prospective serologic cohort study in Hong Kong showed that seroconversion to pdm/09 virus is broadening the serological cross-reactivity in humans to other swine influenza viruses, including EA-like viruses.^15^ Gerloff *et al*.^16^ recently reported higher titers of antibodies against pdm/09 virus and the EA-like H1 viruses in individuals whose professions involve contact with pigs than in a control group without pig contact, and a recent serological survey of swine workers during swine influenza monitoring programs in Italy confirmed this finding.^35^ Although cross-reactivity between pdm/09 virus and EA-like viruses was also detected in pigs, experimental infection with EA-like H1N1 virus has been reported to protect pigs from infection with pdm/09 virus even in the absence of detectable HI cross-reactive antibodies, suggesting that other immune effectors, not necessarily related to HA, can contribute to the cross-protective immunity induced by live viruses.^17^,^36^

Our use of the MVA vector allowed us to better define the role of HA in eliciting cross-protective immunity in mice and thus to determine the extent to which serologic responses correlate with the protection from viral challenge. Mice immunized with MVA-HA-CA/09 were protected from pulmonary challenge with NJ/76 and the EA-like viruses sw/It/02, sw/It/07, sw/It/08, and serological analysis performed with pre-challenge sera showed considerable antigenic cross-reactivity, as revealed by HI assays. By contrast, the absence of cross-reactive HI antibodies specific to sw/IA/30 and sw/It/99 viruses was responsible for the lack of protection against challenge with these viruses. Overall, all mice immunized with MVA-HA-CA/09 were susceptible to i.n. infection with the different challenge viruses used here, with detectable viral titers in the lungs by day 2 post-challenge. However, reduced pulmonary viral loads were evident on day 4 post-challenge for some of these viruses, and this reduction was related to the levels of HI cross-reactive antibodies measured in the sera of the immunized mice before the viral challenge, strongly indicating that the immune protection was dependent on the antigenic similarity of the H1 HAs. This finding is further supported by the sequence identity of the antigenic sites. In particular, comparison of the amino acid sequences of the HAs of NJ/76 and sw/IA/30 viruses showed a high degree of similarity in the antigenic sites, with the exception of one amino acid substitution within the Sa antigenic site (G172E) that might be responsible for the different cross-reactivity of these two viruses to CA/09 virus. The EA-like viruses sw/It/02, sw/It/07, and sw/It/08 are evolutionary and antigenically related, and therefore, they too showed a high degree of similarity at the Sa antigenic site with CA/09 virus, providing an explanation for the HI cross-reactivity and protection of immunized mice from challenges with EA-like viruses. Of note, the lack of HI cross-protective antibodies to sw/It/99 virus was related to the presence of several different amino acid residues in its antigenic sites compared with those in CA/09 virus. Thus, the common protective epitopes between the HA of CA/09 virus and those of the sw/It/02, sw/It/07, sw/It/08, and NJ/76 viruses were responsible for the disease outcome after viral challenge observed in the vaccinated mice. Importantly, the sequences of the antigenic site Sa of the cross-reactive sw/It/02, sw/It/07, and sw/It/08 viruses were also more frequently seen in the H1N1 swine viruses circulating over 13 years in Europe, as compared to those of the non-cross-reactive sw/IA/30 and sw/It/99 viruses.

Our data derived from this mouse model suggest that the immunity acquired by humans during the natural circulation of the pdm/09 virus would reduce the zoonotic risk arising from the majority of the EA-like H1N1 swine viruses. However, it is important to consider that extrapolation of the results obtained through the MVA-based immunization into natural infection could pose some important limitations, due to differences in the antibody repertoires and levels of HI and MN antibodies elicited by mice immunization or natural infection in humans. Indeed, studies aimed at evaluating cross-protective immunity to HA variants of influenza virus in mice immunized with single vectored HAs would help to determine the extent of cross-reactivity related only to the HA protein, thus avoiding immune cross-reactivity related to the NA glycoprotein or the internal viral proteins. Although our experiments were performed with a small number of viruses representative of the CS and EA avian-like swine lineages of viruses circulating in Italy, our results clearly highlight the differences in immune cross-reactivity between the HAs of these swine viruses and CA/09 virus. Using this experimental approach, we should be able to assess the potential risk to human populations that is associated with the introduction of new HA genes into the influenza virus gene pool, which, in turn, may help us to define optimal strategies for immune prophylaxis.

## Supporting Information

**Figure S1.** Alignment of the HA antigenic sites of European H1N1 swine viruses isolated since 1995. 130 amino acids sequences (H1 open reading frame numbering) were retrieved in GenBank database and aligned to the CA/09 HA sequence. Amino acid residue changes in major antigenic sites, as defined by Brownlee and Fodor,^22^ are shown in boxes.

**Table S1.** Antigenic characterization of influenza viruses isolated from Italian pigs.
