# Supplementary figures and images for: Modified vaccinia virus Ankara expressing the hemagglutinin of pandemic (H1N1) 2009 virus induces cross-protective immunity against Eurasian ‘avian-like’ H1N1 swine viruses in mice
Source: Influenza Other Respir Viruses. 2013 Dec 23;8(3):367–75. doi: 10.1111/irv.12221 (PMC4181486; doi:10.1111/irv.12221)

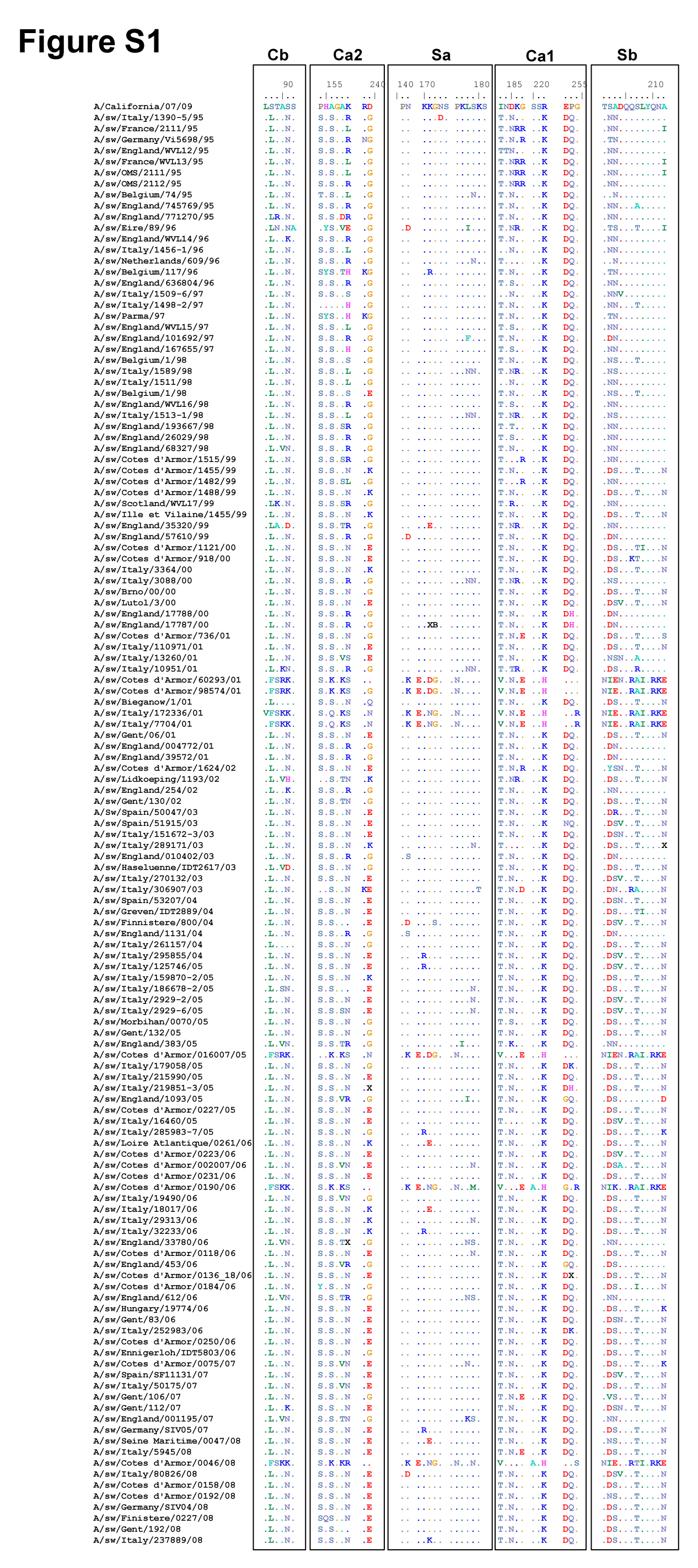

Supplement: Supplementary file 1 — Figure S1. Alignment of the HA antigenic sites of European H1N1 swine viruses isolated since 1995. 130 amino acids sequences (H1 open reading frame numbering) were retrieved in GenBank database and aligned to the CA/09 HA sequence. Amino acid residue changes in major antigenic sites, as defined by Brownlee and Fodor,22 are shown in boxes. [file irv0008-0367-SD1.docx]

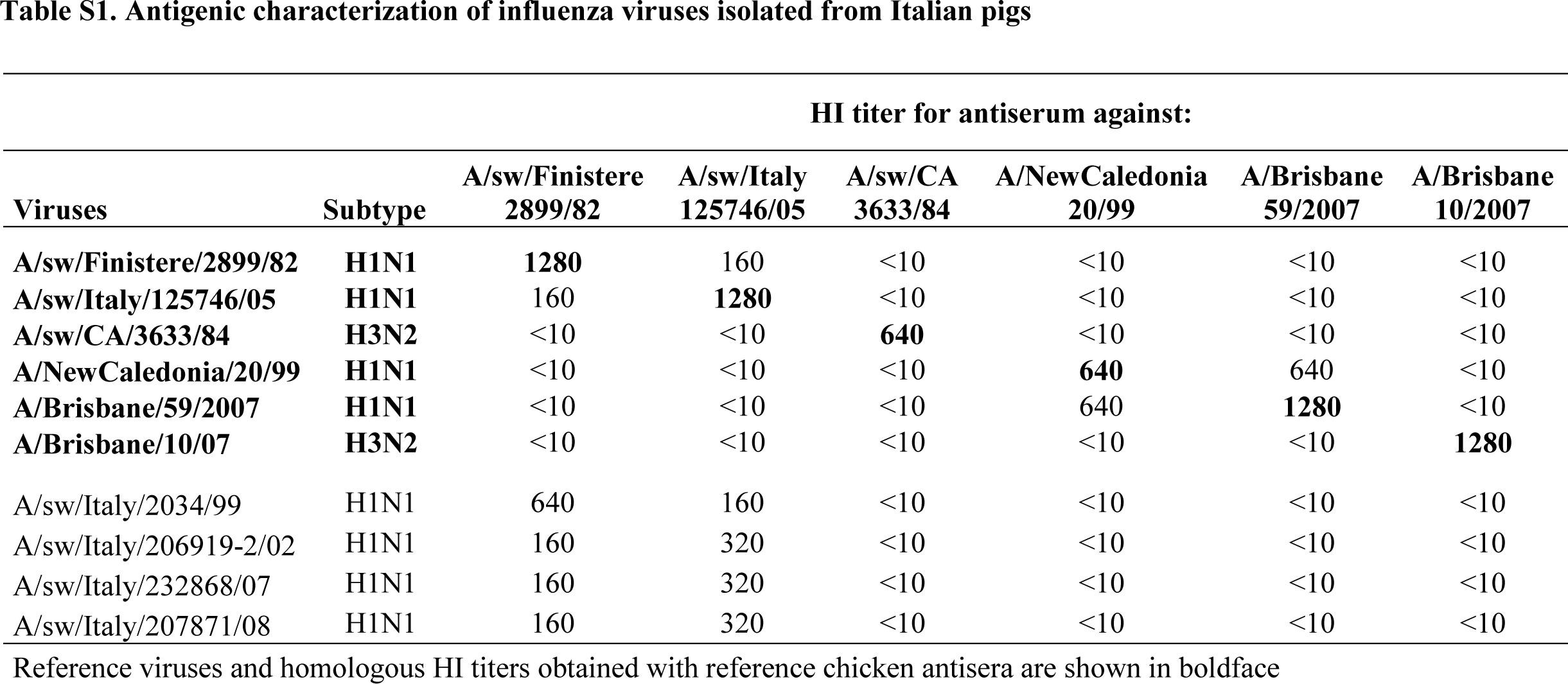

Supplement: Supplementary file 2 — Table S1. Antigenic characterization of influenza viruses isolated from Italian pigs. [file irv0008-0367-SD2.docx]
